# Special Issue “Machine Learning Applications in Bioinformatics and Biomedicine: 3rd Edition”

**DOI:** 10.3390/ijms27156664

**Published:** 2026-07-26

**Authors:** Hao Lyu

**Affiliations:** School of Life Science and Technology, University of Electronic Science and Technology of China, Chengdu 610054, China; hao.lyu@uestc.edu.cn

Machine learning is reshaping bioinformatics and biomedicine by enabling the analysis of high-dimensional molecular, clinical, imaging, and network-based data. With the rapid accumulation of genomic, transcriptomic, proteomic, metabolomic, imaging, and electronic health-related datasets, machine learning has become an important approach for disease biomarker discovery, clinical risk prediction, multi-omics integration, drug target identification, biomedical image analysis, and network-based biological interpretation [1,2,3,4]. In parallel, recent advances in biological artificial intelligence, including foundation and generalist models, are further expanding the scope of computational biology from task-specific prediction toward broader representation learning and cross-domain biological reasoning [5].

This Special Issue, “Machine Learning Applications in Bioinformatics and Biomedicine: 3rd Edition,” comprises ten contributions that reflect recent progress in this rapidly developing field. The published papers cover a broad range of biomedical scenarios, including cancer diagnosis and prognosis, ovarian cancer metabolomics, chronic inflammation-associated cancer susceptibility, Alzheimer’s disease pathology, respiratory disease diagnosis, schizophrenia risk stratification, rare disease genotype–phenotype analysis, and network pharmacology. Collectively, these contributions highlight advances in algorithm development, feature selection, interpretable modeling, multimodal data integration, and automated biomedical analysis platforms. This editorial summarizes the core content and major scientific contributions of these studies from the perspective of the Guest Editor.

The identification of disease biomarkers from genomic and transcriptomic data remains one of the most active areas in biomedical machine learning. Several of the contributions focused on cancer classification, gene selection, and prognostic modeling. Ghuriani et al. (Contribution 1) proposed XGB-BIF, an XGBoost-driven biomarker identification framework for detecting gastric, breast, and lung cancers using human genomic data. By combining XGBoost-based feature selection with classical machine learning classifiers, including support vector machines, logistic regression, and random forest, the framework achieved strong classification performance and identified cancer-associated genes with potential diagnostic relevance. The integration of SHAP, LIME, pathway enrichment, and survival analysis further enhanced the interpretability and translational value of the identified biomarkers. Alkamli and Alshamlan (Contribution 2) introduced GNR, a genetic-embedded nuclear reaction optimization algorithm combined with an F-score filter for gene selection in cancer classification. By improving the exploration and exploitation capability of nuclear reaction optimization through a genetic uniform crossover mechanism, GNR selected compact gene subsets and achieved high classification accuracy across multiple microarray cancer datasets. Zhou et al. (Contribution 3) developed a glycosyltransferase-related prognostic model for clear cell renal cell carcinoma. Through the systematic comparison of 117 machine learning algorithm combinations, the authors established the Glycosyltransferases Risk Score model, which stratified patients into distinct prognostic groups and provided insights into tumor mutation burden, immune microenvironment, immunotherapy response, and key genes such as TYMP and GCNT4. Together, these studies demonstrate the value of machine learning in extracting informative molecular signatures from high-dimensional cancer data and supporting precision oncology.

Multi-omics integration and metabolomics-based disease diagnosis are also important directions represented in this Special Issue. Integrative machine learning has become a central strategy for combining heterogeneous biological and medical data, yet it remains challenged by differences in data scale, sparsity, missingness, and modality-specific noise [4,6]. Tokareva et al. (Contribution 4) developed a machine learning framework for ovarian cancer diagnostics using plasma lipidomics and metabolomics. By integrating HPLC-MS-based lipidomics and NMR-based metabolomics from malignant, benign, and healthy samples, the study systematically evaluated different feature selection strategies and machine learning architectures. The results showed that CNN and XGBoost models achieved strong performance in different diagnostic tasks, highlighting the potential of metabolic and lipidomic signatures for liquid biopsy-based ovarian cancer detection. Hong et al. (Contribution 5) applied an automated machine learning framework to integrate plasma proteomics, post-translational modifications, and metabolomics for schizophrenia risk stratification. Their analysis showed that multi-omics integration improved classification performance compared with single-omics models and identified immune- and coagulation-related molecular events, including immunoglobulin carbamylation and coagulation factor oxidation, as potential biomarkers. These studies show that machine learning can help integrate heterogeneous omics layers and reveal disease-associated molecular patterns that may be missed by single-omics analyses.

Interpretable and biologically informed machine learning represents another important theme of this Special Issue. As machine learning models become more widely used in biomedical and clinical contexts, interpretability is no longer merely a technical preference but an essential requirement for model trust, biological insight, and downstream validation [7]. Cao et al. (Contribution 6) presented FR-BINN, a biologically informed neural network framework for biomarker discovery and pathway analysis in chronic inflammatory diseases. By incorporating Fenton reaction-related biological priors into a neural network architecture and combining multiple interpretability methods with large language model-assisted semantic reasoning, FR-BINN identified key genes and biological processes associated with cancer-prone and non-cancer-prone chronic inflammatory diseases. This study provides a valuable example of how biological knowledge can be embedded into deep learning models to improve both predictive performance and mechanistic interpretability. Serrano-Gonzalo et al. (Contribution 7) investigated clinical variability and genotype–phenotype correlations in Spanish patients with type 1 Gaucher disease, with a focus on non-c.[1226A>G];[1448T>C] genotypes. By combining registry-based clinical analysis with logistic regression and decision tree models, the authors identified genotype-associated differences in skeletal involvement and Parkinsonism risk. This work highlights the potential of predictive modeling in rare disease research, where clinical heterogeneity often limits genotype-based risk assessment.

Machine learning is also advancing biomedical image analysis and clinical decision-support systems. Deep learning has achieved a strong performance in many medical imaging tasks, but clinical translation still requires careful validation, robust generalization, and efficient annotation strategies [8,9]. Barczánfalvi et al. (Contribution 8) developed a weakly supervised deep learning pipeline for automated segmentation and morphometric analysis of thioflavin-S-stained amyloid deposits in Alzheimer’s disease brains and age-matched controls. By integrating image preprocessing, class activation mapping, synthetic data generation, and U-Net-based segmentation, the study achieved robust amyloid plaque segmentation without requiring dense pixel-level annotations. This contribution demonstrates the value of weakly supervised learning in histopathology, where manual annotation is expensive and time-consuming. Abdullah et al. (Contribution 9) presented a multimodal AI framework for multiclass lung disease diagnosis from respiratory sounds, combined with simulated biomarker fusion and personalized medication recommendation. The framework classified multiple respiratory disease categories from audio recordings and explored a proof-of-concept prescription recommendation module. Although further validation using real clinical records is required, this study illustrates the potential of multimodal AI systems for future respiratory care and clinical decision support.

Automated computational platforms are essential for improving the reproducibility and efficiency of biomedical data analysis. In network pharmacology and systems biology, computational frameworks can help connect genes, compounds, diseases, and biological pathways within interpretable molecular networks [10]. Ping et al. (Contribution 10) introduced NeXus, an automated platform for network pharmacology and multi-method enrichment analysis. NeXus integrates network construction with ORA, GSEA, and GSVA, enabling efficient analysis of gene–compound–plant relationships and the generation of publication-quality visualizations. The platform reduced manual workflow time and improved the integration of multi-layer biological relationships. This contribution provides a practical computational resource for network pharmacology, systems biology, and drug discovery research.

Taken together, the contributions in this Special Issue demonstrate the broad and growing impact of machine learning in bioinformatics and biomedicine. These studies move beyond simple disease classification and emphasize feature selection, multi-omics integration, biological interpretability, external validation, automated analysis, and translational relevance. At the same time, they also highlight continuing challenges, including limited sample sizes, dataset heterogeneity, the need for prospective validation, and the importance of connecting computational predictions with experimental and clinical evidence. Future progress in this field will depend on the development of more robust, interpretable, and biologically grounded machine learning frameworks that can bridge molecular discovery and clinical application.

The Guest Editor would like to thank all authors for their valuable contributions to this Special Issue and all anonymous reviewers for their careful evaluation, constructive comments, and efforts in improving the quality of the published manuscripts.
